# Epidemiological characteristics of primary spinal osseous tumors in Eastern China

**DOI:** 10.1186/s12957-017-1136-1

**Published:** 2017-04-04

**Authors:** Zhenhua Zhou, Xudong Wang, Zhipeng Wu, Wending Huang, Jianru Xiao

**Affiliations:** Department of Orthopaedic Oncology, Changzheng Hospital,, The Second Military Medical University, No.415, Fengyang Road, Shanghai, 200003 China

**Keywords:** Spine, Primary osseous tumors, Epidemiology

## Abstract

**Background:**

Primary spinal osseous tumors are rare, yet they represent a difficult treatment paradigm because of the complexities of tumor resection and significant resistance to chemotherapy and radiation therapy. The geographic distribution of primary spinal osseous tumors throughout the world appears to be quite variable, with a very low incidence reported in Asian countries.

**Methods:**

Data on 1209 cases of primary spinal osseous malignant and benign tumor cases diagnosed during the 20-year period of 1995 through 2015 in eastern China were analyzed**.**

**Results:**

In 780 cases (64.5%), the lesion was benign and in 429 (35.5%) was malignant. The commonest primary malignant tumors were chordoma (9.8% of all cases) followed by plasma cell myeloma (8.5% of all cases). The most common benign tumor was hemangioma (28.1% of all cases) followed by giant cell tumor of bone (15.7% of all cases) and osteoblastoma (4.4% of all cases). The benign tumors affected men in 33.8% of cases and women in 30.7% of cases, the malignant tumors affected men in 23.7% of cases and women in 11.8%. The mean age (mean ± SD) in the benign group was 34.7 ± 19.8 years and in the malignant group was 47.4 ± 16.5 years. Related symptoms were pain (54.4%), radiculopathy (12.9%), cord compression (9.2%), mass (5.7%), pathological fracture (4.7%), deformity (2.1%), and weight loss (1.9%). The anatomical locations included almost every vertebra of the spine. The thoracic spine (38.1%) was the most common location of the tumors, followed by the cervical spine (27.4%) and lumbar spine (18.4%).

**Conclusions:**

Compared with other similar series reported in the literature from the other countries, our results obtained in a developing country were different in some degree. This large series of primary spinal osseous tumors may reflect fairly well their real incidence and provide a sufficiently detailed perspective on epidemiologic studies of primary spinal osseous tumors in eastern China.

**Electronic supplementary material:**

The online version of this article (doi:10.1186/s12957-017-1136-1) contains supplementary material, which is available to authorized users.

## Background

Primary spinal osseous tumors are uncommon; previous reports showed that spinal osseous tumors were comprising 6 ~ 10% of all bone tumors [1–5]. Little was known about the etiology of spine osseous tumors. Published case reports focused on spine neoplasm usually had a limited case number, and few reports described epidemiological characteristics of spine osseous tumors. However, it is important to understand the etiology of spinal osseous tumors and information regarding the epidemiology of primary spine osseous tumors.

The present study performed an epidemiological analysis of 1209 consecutive tumors of osseous spine registered in 3 collaborating state bone tumor database (Eastern China) between 1995 and 2015. To our knowledge, the present study is the first report of epidemiological data concerning spinal osseous tumors in Asian population. Our findings showed that epidemiological features of primary spinal osseous tumors in xanthoderm, with respect to relative frequency and distribution of the various histologic types, as well as the clinical data, and compare the results with other epidemiological findings from different geographic locations around the world, which could provide valuable clues for epidemiology of primary spine osseous tumors in Asian.

## Methods

### Data sources

Data for this study were obtained from Bone Tumors and Nervous System Tumors Biobank of Shanghai (BT&NSTBS), Bone Tumor Sample Databases and Digital Information Platform of Shanghai (BTSD&DIPS), and Shanghai Biobank Network of Common Human Tumor Tissue (SBNCHTT). One thousand two hundred nine cases of spinal osseous tumors registered in abovementioned 3 database between 1995 and 2015 were selected for this study. Data collected for each patient included personal information such as name, age, sex, anatomical site of the tumor, and clinical and histological diagnoses. In the case of recurrent tumors, the histological appearance of the original and the recurrent tumors was compared and was considered as only one case. The diagnoses were re-evaluated according to the criteria suggested for the 2013 WHO histological classification [6].

### Statistics

SPSS17.0 software (SPSS Inc., Chicago, IL) was used for statistical analysis of experimental data. Descriptive statistics was performed to calculate the frequency and percentages of variables mentioned before. Age was stratified into various groups at 10-year intervals.

## Results

**Fig. 1:**
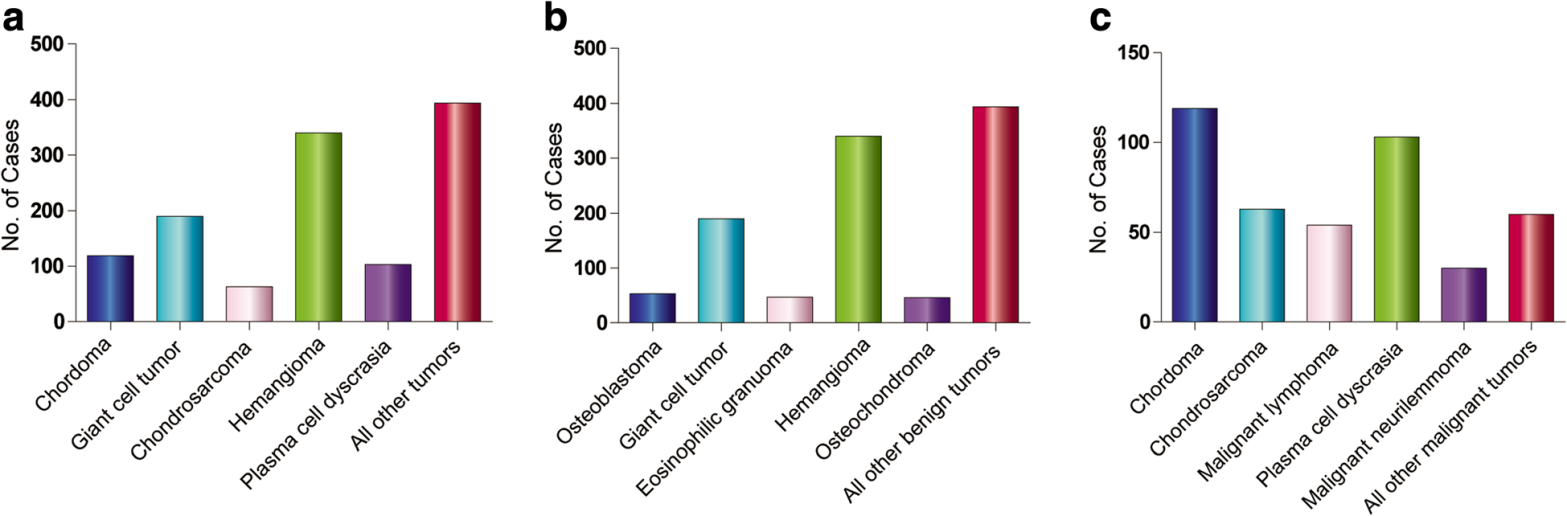
Distribution of primary spine osseous tumors. Distribution of **a** all primary spine osseous tumor, **b** benign spine osseous, and **c** malignant spine osseous tumor cases by histological type

The histological types of the spinal osseous tumors are listed in Table 1 and Fig. 1. Of these, 64.5% (780 cases) were benign and 35.5% (429 cases) were malignant, The most common histological type of benign tumors was hemangioma accounting for 28.1% of all tumors (340 cases), followed by giant cell tumor (15.7%; 190 cases), osteoblastoma (4.4%; 53 cases), aneurysmal bone cyst (2.9%; 35 cases), eosinophilic granuloma (3.9%; 47 cases), osteochondroma (3.8%; 46 cases), solitary bone cyst (2.1%; 25 cases), osteoid osteoma (1.4%; 17 cases), fibroma (1.3%; 16 cases), lipoma (0.6%; 7 cases), and fibrous dysplasia (0.3%; 4 cases). Of malignant tumors, chordoma was the most common malignant tumor (9.8% of all tumors, 119 cases), followed by plasma cell myeloma multiple myeloma (8.5%,103 cases), chondrosarcoma (5.2%, 63 cases), malignant lymphoma (4.5%; 54 cases), malignant neurilemmoma (2.5%; 30 cases), primitive neural ectodermal tumor (PNET)\Ewing’s sarcoma (1.4%; 17 cases), malignant fibrous histiocytoma (1.3%; 16 cases), osteosarcoma (1.1%; 13 cases), and other sarcomas such as angiosarcoma, fibrosarcoma, liposarcoma, and leiomyosarcoma (less than 1%).

**Table 1:** Frequency, age, and gender distribution of primary spine osseous tumors

Type of tumor	Number (%)		Male		Female		Male vs female	Age range (years)	Mean ± SD (age)		
	No.	%^a^	No.	%^a^	No.	%^a^			Male	Female	Total
Benign tumors	780	64.5	409	33.8	371	30.7	52.4 vs 47.6%	9–79	33.7 ± 19.2	35.9 ± 20.6	34.7 ± 19.8
Hemangioma	340	28.1	174	14.4	166	13.7	51.2 vs 48.8%	12–79	47.1 ± 14.3	51.5 ± 12.9	49.2 ± 13.7
Giant cell tumor	190	15.7	91	7.5	99	8.2	47.9 vs 52.1%	15–66	34.5 ± 11.0	32.9 ± 12.1	33.6 ± 11.5
Eosinophililc granuloma	47	3.9	36	3.0	11	0.9	76.6 vs 23.4%	10–56	23.7 ± 14.3	29 ± 17.6	23.7 ± 14.7
Osteoblastoma	53	4.4	28	2.3	25	2.1	52.8 vs 47.2%	9–54	32.6 ± 10.5	26.5 ± 15.0	28.7 ± 13.2
Fibroma	16	1.3	10	0.8	6	0.5	62.5 vs 37.5%	26–76	41.6 ± 18.2	50.5 ± 15.0	44.9 ± 17.1
Osteoid osteoma	17	1.4	8	0.7	9	0.7	47.1 vs 52.9%	16–67	31.2 ± 22.3	39.0 ± 16.8	34.6 ± 19.8
Osteochondroma	46	3.8	28	2.3	18	1.5	60.9 vs 39.1%	13–64	34.0 ± 16.6	38.7 ± 20.8	36.1 ± 18.4
Solitary bone cyst	25	2.1	8	0.7	17	1.4	32.0 vs 68.0%	15–64	29.5 ± 15.2	48.8 ± 11.9	41.1 ± 16.2
Lipoma	7	0.6	5	0.4	2	0.2	71.4 vs 28.6%	23–63	41.8 ± 16.5	31.0 ± 11.3	38.7 ± 15.2
Aneurysmal bone cyst	35	2.9	19	1.6	16	1.3	54.3 vs 45.7%	10–62	25 ± 12.5	36.4 ± 15.3	30.4 ± 14.8
Fibrous dysplasia	4	0.3	2	0.2	2	0.2	50.0 vs 50.0%	25–32	29.5 ± 3.5	27 ± 2.8	28.3 ± 3.0
Malignant tumors	429	35.5	286	23.7	143	11.8	66.7 vs 33.3%	8–81	48.3 ± 16.7	46.3 ± 16.3	47.4 ± 16.5
PNET/ Ewing’s sarcoma	17	1.4	9	0.7	8	0.7	52.9 vs 47.1%	11–46	26 ± 13.3	25.8 ± 7.9	25.9 ± 10.9
Chordoma	119	9.8	79	6.5	40	3.3	66.4 vs 33.6%	27–81	56.6 ± 15.0	53.3 ± 13.3	55.5 ± 14.1
Malignant fibrous histiocytoma	16	1.3	11	0.9	5	0.4	68.8 vs 31.2%	29–67	51 ± 14.5	50.7 ± 1.9	50.9 ± 11.3
Liposarcoma	1	0.0	1	0.0	0	0.0	NA	44	44	NA	44
Osteosarcoma	13	1.1	5	0.4	8	0.7	38.5 vs 61.5%	14–60	40.2 ± 21.0	25.8 ± 11.7	33.6 ± 18.2
Angiosarcoma	10	0.8	7	0.6	3	0.2	70.0 vs 30.0%	36–73	54.3 ± 11.3	45.0 ± 7.8	51.5 ± 10.9
Malignant neurilemmoma	30	2.5	15	1.2	15	1.2	50.0 vs 50.0%	8–71	40.4 ± 16.3	50.3 ± 12.1	45.3 ± 15.0
Plasma cell myeloma	103	8.5	75	6.2	28	2.3	72.8 vs 27.2%	10–76	49.8 ± 15.3	57.2 ± 12.6	52.0 ± 14.8
Malignant lymphoma	54	4.5	30	2.5	24	2.0	55.6 vs 44.4%	10–77	47.2 ± 21.0	46.5 ± 18.7	46.9 ± 19.6
Leiomyosarcoma	1	0.0	1	0.0	0	0.0	NA	55	55	NA	55
Chondrosarcoma	63	5.2	52	4.3	11	0.9	82.5 vs 17.5%	20–68	46.1 ± 14.5	37.3 ± 14.8	44.0 ± 14.7
Fibrosarcoma	2	0.2	1	0.0	1	0.0	50.0 vs 50.0%	30–61	61	30	45.5 ± 21.9
Total	1209	100	695	57.5	514	42.5	57.5 vs 42.5%	8–81	39.1 ± 16.9	39.6 ± 16.7	39.3 ± 16.8

*NA* not applicable

^a^% in all tumors

Of the benign tumors, 20.5% were situated in the cervical spine, 26.1% in the thoracic spine, 12.0% in the lumbar spine, and 5.4% in the sacral spine. Of the malignant tumors, 6.9% were situated in the cervical spine, 12.0% in the thoracic spine, 6.3% in the lumbar spine, and 10.7% in the sacral spine. Of all tumors, 27.4% were situated in the cervical spine, 38.1% in the thoracic spine, 18.4% in the lumbar spine, and 16.1% in the sacral spine (Table 2).

**Table 2:** Distribution of spine osseous tumors by location

Type of tumor	Cervical spine		Thoracic spine		Lumbar spine		Sacral spine		Totals	
	No.	%	No.	%	No.	%	No.	%	No.	%
Benign tumors	257	20.5	328	26.1	151	12.0	68	5.4	804(780)^a^	64.0
Hemangioma	104	8.3	149	11.7	86	6.8	11	0.9	350(340)^a^	27.9
Giant cell tumor	44	3.5	87	6.9	23	1.8	36	2.9	190	15.1
Eosinophililc granuoma	20	1.6	22	1.6	5	0.4	0	0.0	47	3.7
Osteoblastoma	17	1.4	30	2.4	5	0.4	1	0.0	53	4.2
Fibroma	17	1.4	5	0.4	5	0.4	3	0.2	30(16)	2.4
Osteoid osteoma	12	1.0	2	0.2	3	0.2	0	0.0	17	1.4
Osteochondroma	21	1.7	12	1.0	11	0.9	2	0.2	46	3.7
Solitary bone cyst	8	0.6	2	0.2	5	0.4	10	0.8	25	2.0
Lipoma	5	0.4	1	0.0	0	0.0	1	0.0	7	0.6
Aneurysmal bone cyst	9	0.7	16	1.3	6	0.5	4	0.3	35	2.8
Fibrous dysplasia	0	0.0	2	0.2	2	0.2	0	0.0	4	0.3
Malignant tumors	87	6.9	151	12.0	80	6.3	134	10.7	452(429) ^a^	36.0
PNET/Ewing’s sarcoma	1	0.0	4	0.3	9	0.7	3	0.2	17	1.4
Chordoma	29	2.3	5	0.4	5	0.4	80	6.4	119	9.5
Malignant fibrous histiocytoma	0	0.0	8	0.6	3	0.2	5	0.4	16	1.3
Liposarcoma	0	0.0	0	0.0	0	0.0	1	0.0	1	0.0
Osteosarcoma	2	0.2	6	0.6	0	0.0	5	0.4	13	1.0
Angiosarcoma	1	0.0	12	1.0	4	0.3	0	0.0	17(10)	1.4
Malignant neurilemmoma	4	0.3	4	0.3	12	1.0	12	1.0	32(30) ^a^	2.5
Plasma cell myeloma	27	2.1	50	4.0	26	2.1	9	0.7	112(103)	8.9
Leiomyosarcoma	0	0.0	0	0.0	0	0.0	1	0.0	1	0.0
Malignant lymphoma	9	0.7	26	2.1	18	1.4	6	0.5	59(54)^a^	4.7
Chondrosarcoma	14	1.1	36	2.9	2	0.2	11	0.9	63	5.0
Fibrosarcoma	0	0.0	0	0.0	1	0.0	1	0.0	2	0.2
Total	344	27.4	479	38.1	231	18.4	202	16.1	1256(1209) ^a^	100

^a^More than two anatomical position involved in the same patient

In our series, most tumors with different histological types showed a similar distribution in males and females, although chordoma (119 cases, M:F = 2:1), plasma cell myeloma (103 cases, M:F = 2.7:1), chondrosarcoma (63 cases, M:F = 4.7:1), and eosinophilic granuoma (47 cases, M:F = 3.3:1) affected more frequently males than females (Table 3). From a total of 1209 bone tumors, the mean age (mean ± SD) was 39.3 ± 16.8 years (range, 8–81 years), 57.5% (695 cases) of the tumors occurred in males and 42.5% (514cases) in females, with a mean age (mean ± SD) of 39.1 ± 16.9 and 39.6 ± 16.7 years. The mean age (mean ± SD) of benign tumors group was 34.7 ± 19.8 years (range, 9–79 years). Of 780 benign tumors, 409 cases (33.8% of all cases) occurred in males and 371 cases (30.7% of all cases) in females (M:F = 1.1:1), with a mean age (mean ± SD) of 33.7 ± 19.2 and 35.9 ± 20.6 years. The mean age (mean ± SD) of malignant tumors group was 47.4 ± 16.5 years (range, 8–81 years). Of 429 malignant tumors, 286 cases (23.7% of all cases) occurred in males and 143 cases (11.8% of all cases) in females, with a mean age (mean ± SD) of 48.3 ± 16.7 and 46.3 ± 16.3 years. The most commonly affected age group for benign tumors was the 31- to 40-year-old group (14.3%; 173 cases), followed by the 51- to 60-year-old group (11.4%; 138 cases), and by the 41- to 50-year-old group (11.2%; 136 cases). The most common age group affected by malignant bone tumors was the 41- to 50-year-old group (7.9%; 95cases), followed by the 61- to 70-year-old group (6.8%; 82 cases). A similar frequency was observed in the 31- to 40-year-old group (6.7%; 81cases) and the 51- to 60-year-old group (6.5%; 79 cases).

**Table 3:** Age distribution of patients with primary spine osseous tumors (years)

Type of tumor	0–10	11–20	21–30	31–40	41–50	51–60	61–70	71–80	81–90	Total no. (%)
	No. (%)	No. (%)	No. (%)	No. (%)	No. (%)	No. (%)	No. (%)	No. (%)	No. (%)	
Benign tumors	14(1.2%)	102(8.4%)	122(10.1%)	173(14.3%)	136(11.2%)	138(11.4%)	76(6.3%)	19(1.6%)	0(0.0%)	780(64.5%)
Hemangioma	1(0.0%)	14(1.2%)	18(1.5%)	62(5.1%)	78(6.5%)	96(7.9%)	53(4.4%)	18(1.5%)	0(0.0%)	340(28.1%)
Giant cell tumor	0(0.0%)	27(2.2%)	50(4.1%)	62(5.1%)	32(2.6%)	11(0.9%)	8(0.7%)	0(0.0%)	0(0.0%)	190(15.7%)
Eosinophililc granuloma	3(0.2%)	23(1.9%)	9(0.7%)	5(0.4%)	2(0.2%)	5(0.4%)	0(0.0%)	0(0.0%)	0(0.0%)	47(3.9%)
Osteoblastoma	6(0.5%)	11(0.9%)	14(1.2%)	11(0.9%)	5(0.4%)	6(0.5%)	0(0.0%)	0(0.0%)	0(0.0%)	53(4.4%)
Fibroma	0(0.0%)	0(0.0%)	4(0.3%)	4(0.3%)	2(0.2%)	2(0.2%)	3(0.2%)	1(0.0%)	0(0.0%)	16(1.3%)
Osteoid osteoma	0(0.0%)	5(0.4%)	3(0.2%)	2(0.2%)	2(0.2%)	3(0.2%)	2(0.2%)	0(0.0%)	0(0.0%)	17(1.4%)
Osteochondroma	2(0.2%)	12(1.0%)	8(0.7%)	9(0.7%)	5(0.4%)	5(0.4%)	5(0.4%)	0(0.0%)	0(0.0%)	46(3.8%)
Solitary bone cyst	0(0.0%)	2(0.2%)	3(0.2%)	5(0.4%)	6(0.5%)	6(0.5%)	3(0.2%)	0(0.0%)	0(0.0%)	25(2.1%)
Lipoma	0(0.0%)	0(0.0%)	2(0.2%)	3(0.2%)	1(0.0%)	1(0.0%)	0(0.0%)	0(0.0%)	0(0.0%)	7(0.6%)
Aneurysmal bone cyst	2(0.2%)	8(0.7%)	9(0.7%)	8(0.7%)	3(0.2%)	3(0.2%)	2(0.2%)	0(0.0%)	0(0.0%)	35(2.9%)
Fibrous dysplasia	0(0.0%)	0(0.0%)	2(0.2%)	2(0.2%)	0(0.0%)	0(0.0%)	0(0.0%)	0(0.0%)	0(0.0%)	4(0.3%)
Malignant tumors	5(0.4%)	17(1.4%)	36(3.0%)	81(6.7%)	95(7.9%)	79(6.5%)	82(6.8%)	32(2.6%)	2(0.2%)	429(35.5%)
PNET/Ewing’s sarcoma	0(0.0%)	5(0.4%)	7(0.6%)	3(0.2%)	2(0.2%)	0(0.0%)	0(0.0%)	0(0.0%)	0(0.0%)	17(1.4%)
Chordoma	0(0.0%)	0(0.0%)	3(0.2%)	17(1.4%)	27(2.2%)	23(1.9%)	32(2.6%)	15(1.2%)	2(0.2%)	119(9.8%)
Malignant fibrous histiocytoma	0(0.0%)	0(0.0%)	2(0.2%)	0(0.0%)	5(0.4%)	6(0.5%)	3(0.2%)	0(0.0%)	0(0.0%)	16(1.3%)
Liposarcoma	0(0.0%)	0(0.0%)	0(0.0%)	0(0.0%)	1(0.0%)	0(0.0%)	0(0.0%)	0(0.0%)	0(0.0%)	1(0.0%)
Osteosarcoma	0(0.0%)	6(0.5%)	2(0.2%)	0(0.0%)	3(0.2%)	2(0.2%)	0(0.0%)	0(0.0%)	0(0.0%)	13(1.1%)
Angiosarcoma	0(0.0%)	0(0.0%)	0(0.0%)	2(0.2%)	3(0.2%)	3(0.2%)	1(0.0%)	1(0.0%)	0(0.0%)	10(0.8%)
Malignant neurilemmoma	1(0.0%)	1(0.0%)	1(0.0%)	9(0.7%)	8(0.7%)	5(0.4%)	4(0.3%)	1(0.0%)	0(0.0%)	30(2.5%)
Plasma cell myeloma	2(0.2%)	0(0.0%)	3(0.2%)	17(1.4%)	26(2.2%)	22(1.8%)	24(2.0%)	9(0.7%)	0(0.0%)	103(8.5%)
Leiomyosarcoma	0(0.0%)	0(0.0%)	0(0.0%)	0(0.0%)	0(0.0%)	1(0.0%)	0(0.0%)	0(0.0%)	0(0.0%)	1(0.0%)
Malignant lymphoma	2(0.2%)	3(0.2%)	6(0.5%)	9(0.7%)	12(1.0%)	8(0.7%)	8(0.7%)	6(0.5%)	0(0.0%)	54(4.5%)
Chondrosarcoma	0(0.0%)	2(0.2%)	11(0.9%)	24(2.0%)	8(0.7%)	9(0.7%)	9(0.7%)	0(0.0%)	0(0.0%)	63(5.2%)
Fibrosarcoma	0(0.0%)	0(0.0%)	1(0.0%)	0(0.0%)	0(0.0%)	0(0.0%)	1(0.0%)	0(0.0%)	0(0.0%)	2(0.2%)
Total	19(1.6%)	119(9.8%)	158(13.1%)	254(21.0%)	231(19.1%)	217(17.9%)	158(13.1%)	51(4.2%)	2(0.2%)	1209(100%)

As the most common presenting symptom, pain was found in 54.4% (658/1209) of patients, affecting 35.0% (273/780) of the benign and 89.7% (385/429) of the malignant tumors. Mass (swelling) was seen in 4.0% (31/780) of benign tumors and 8.9% (38/429) of malignant tumors. One hundred fifty-six patients (12.9%) had the symptoms of radiculopathy. Of all cases, 9.2% (111/1209) of patients had signs of subtotal or complete cord compression. The cord compression symptoms included motor weakness (8.2%, 99/1209), sphincter dysfunction (0.7%, 8/1209), and paraplegia (0.3%, 4/1209). Pathological fracture was found in 4.7% (57/1209) of patients. Other symptoms included deformity (2.1%, 25/1209) and weight loss (1.9%, 23/1209) (Table 4).

**Table 4:** First presenting features when diagnosed with primary spine osseous tumors

	Total no. of case	Pain	Mass (swelling)	Radiculopathy	Motor weakness	Sphincter dysfunction	Paraplegia	Pathological fracture	Deformity	Weight loss
Benign tumors	780	273(35.0%)	31(4.0%)	72(9.2%)	45(5.8%)	0	0	23(2.9%)	4(0.5%)	7(0.9%)
Hemangioma	340	18	2	1	0	0	0	0	0	1
Giant cell tumor	190	169	11	65	43	0	0	21	3	2
Eosinophililc granuloma	47	35	17	4	1	0	0	0	0	1
Osteoblastoma	53	2	0	0	0	0	0	0	0	0
Fibroma	16	4	0	0	0	0	0	0	0	0
Osteoid osteoma	17	3	0	0	0	0	0	0	0	1
Osteochondroma	46	13	0	0	0	0	0	0	0	1
Solitary bone cyst	25	2	1	0	0	0	0	0	0	0
Lipoma	7	0	0	0	0	0	0	0	0	0
Aneurysmal bone cyst	35	25	0	1	1	0	0	1	0	1
Fibrous dysplasia	4	2	0	1	0	0	0	1	1	0
Malignant tumors	429	385(89.7%)	38(8.9%)	84(19.9%)	54(12.6%)	8(1.9%)	4(0.9%)	34(7.9%)	21(4.9%)	16(3.7%)
PNET/Ewing’s sarcoma	17	16	1	0	0	0	0	1	0	0
Chordoma	119	101	15	43	35	7	2	12	6	3
Malignant fibrous histiocytoma	16	13	10	0	0	0	0	1	0	0
Liposarcoma	1	1	0	1	0	0	0	1	0	0
Osteosarcoma	13	13	2		0	0	0	0	0	0
Angiosarcoma	10	9	1	1	0	0	0	0	0	0
Malignant neurilemmoma	30	28	0	28	15	1	0	0	0	0
Plasma cell myeloma	103	92	0	7	2	2	2	19	15	13
Leiomyosarcoma	54	51	7	3	1	0	0	0	0	0
Malignant lymphoma	1	1	0	0	0	0	0	0	0	0
Chondrosarcoma	63	58	0	0	0	0	0	0	0	0
Fibrosarcoma	2	2	2	1	1	0	0	0	0	0
Total	1209	658(54.4%)	69(5.7%)	156(12.9%)	99(8.2%)	8(0.7%)	4(0.3%)	57(4.7%)	25(2.1%)	23(1.9%)

In our series, 398 cases with malignant tumors and 187 cases with benign tumors undergone surgery. Two hundred seventy-one cases with malignancies received chemotherapy. Thirty-four cases with malignant tumors were given radiotherapy. And other treatments were performed to 6 malignant cases and 35 benign cases respectively (Additional file 1: Table S1).

## Discussion

Primary bone tumors of the spine are representing only less than 10% of all bone neoplasms. Available reports on the epidemiologic features of primary osseous spine tumors were mostly among Americans and Europeans [7–11]. To our knowledge, the present report represents epidemiologic features based the largest series of spinal osseous tumors from Asian. However, there are some differences of epidemiological characteristics in our study and previous reports (Table 5) [8, 10]. In Kelley’s series [10], 23.02% tumors were benign and 76.98% tumors were malignant, plasma cell myeloma was the most common primary malignant tumor, accounting for 26%, followed by chordoma (22.22%) and osteosarcoma (9.52%). Osteoblastoma (5.56%) was the most common benign tumor, followed osteoclastoma (0.79%) and aneurysmal bone cyst (0.79%). In our series, benign tumors comprised 64.5% of primary tumors. The most frequent benign was hemangioma (28.1%), followed by giant cell tumor (15.7%) and osteoblastoma (4.4%). The most frequent malignant was chordoma (9.8%), followed by plasma cell myeloma (8.5%) and chondrosarcoma (5.2%). These findings were not consistent with results from previous studies based in Caucasian, which would be influenced by geographical and racial variations in different reports.

**Table 5:** World literature of population-based studies on the epidemiology of primary spine osseous tumor

	This study	Simon P. Kelley	S. Boriani
Author’s country	China	UK	Italy
Year of publication	2016	2007	1995
Period of study	1995–2015 (20 years)	1958–2000 (42 years)	1946–1992 (46 years)
No. of cases	1209	127	366
Gender	Male: 695 Female:514	Male: 66 Female:61	Not reported
Predominant presenting symptom	Pain	Pain	Not reported
Percentage of benign tumors	64.5%	22.8%	56.8%
Percentage of malignant tumors	35.5%	77.2%	43.2%
Most common type of benign tumors	Hemangioma	Osteoblastoma (7/127)	Eosinophilic granuloma
Most common type of malignant tumors	Chordoma	Plasma cell myeloma	Plasma cell myeloma
Most common segment of spine affected	Thoracic spine (479/1256)^a^	Thoracic spine (48/127)	Lumbar spine (181/366)
Mean age at presentation (range)	39.3 ± 16.8 (range 8–81 years)	42 (range 7–76 years)	Not reported

^a^More than two anatomical position involved in the same patient

As the most frequent benign primary spine osseous tumor in present study, hemangioma was accounting for approximately one third of all primary tumors. During the past 20 years (1995–2015), 340 (28.1% of all cases) cases of spinal hemangioma were diagnosed, and 350 vertebrae were involved. This incidence of spinal hemangioma was significantly higher than that in other country [12], which suggested different epidemiologic features of spinal hemangioma in Asians. Giant cell tumor (GCT) of bone was considered a benign osteolytic tumor with variable aggressiveness and accounted for approximately 5% of all bone tumors [4, 13, 14]. In our study, GCTs accounted for 15.7% of spine osseous lesions, this incidence was markedly higher than that reported in other studies [15–19]. Almost of GCTs occurred in the second to fourth decades, with a slight female predilection (M:F = 1:1.1) in the present series. The female predilection of GCT is supported by the earlier reports [17, 20–22]. Furthermore, GCTs also showed predilection for the thoracic vertebra of spine in our study, which is not in accordance with other reports [19, 23]. Osteoblastoma was the third most common benign tumor in our study, with a relative frequency of 4.4% which was higher compared with the previous report [24]. In Herman M. Kroon’s and Richard A. Mcleod’s reports [25, 26], osteoblastoma obviously affected more males than females (64:34 and 87:36 respectively). In our study, we only observed a slight male predilection (M:F = 1:1.1). The most involved location was the thoracic spine, which is consistent with those reported from Europe and America [27, 28].

Chordoma was the most common malignant tumor which occurred more in males than in females in the present study. We found a significant predilection for males (M:F = 79:40), in agreement with reports by S Boriani (M:F = 37:15) [29], Silvia Stacchiotti (M:F = 91:47) [30], and Johannm Bjornsson (M:F = 27:13) [31]. It is worth noting that female preponderance (17:22) of chordoma was reported in Sweden [32]. In our series, chordomas were typically seen in adults and elderly, tended to occur in the sacrum (9.5%, 119 cases), which is consistent with the previous reports. In the present study, plasma cell myeloma accounted for 8.5% (103 cases) of all cases, representing the second most frequent primary malignant osseous tumor of spine, and often occurring in thoracic spine (4.0%, 50 vertebrae were involved). All plasma cell myeloma cases were found in the 4th–7th decade of life with a male predominance (M:F = 2.7:1). Previous studies reported that chondrosarcoma accounted for 3–12% of all spine primary tumors [33–36]. In S Boriani’s report, the lumbar spine was the most frequently involved location (15/22, 68%) [33]. However, in the present study, thoracic spine was the most common location involved with chondrosarcoma (2.9%, 36 cases), and only 2 cases were observed in the lumbar spine. Most patients commonly encountered chondrosarcoma after the age of 20, with a great male predilection (M:F = 4.7:1).

Surgery is the first choice for the pain and neurological symptoms caused by spine tumors. Surgery can completely or partly alleviate tumor compression to the spinal cord, establish a tumor-free solid spine and relieve pain. In primary malignant tumors of the spine, total/partial laminectomy, total/partial vertebral body resection, and piecemeal resection and curettage, in addition to the surgical procedures described above, can be used. Chemotherapy and radiotherapy after surgery were used to reduce the risk of the cancer relapse or shrinks some malignant tumor such Ewing’s sarcoma that cannot be completely removed with surgery. Many benign silent (no symptom) tumors such as hemangioma were found in health checkup, and most of them did not receive any treatment. Although the WHO defines GCT as a benign osteolytic neoplasm, GCT displays the characteristics of both malignant and benign tumors and actually represents a benign tumor with the potential of malignancy [37–39]. In our series, most cases of GCT undergone total spondylectomy, vertebrectomy, piecemeal resection or curettage. Some cases of GCT received other therapy such as bisphosphonate treatment after surgery.

## Conclusions

Our study results represent data on the epidemiology of spinal osseous tumors in a large population of patients. We think that these data may reflect the epidemiological features of spine osseous tumors in Eastern China. For geographical and racial variations, the incidence of these neoplasms in a developing country is partly different from that found in other countries. There were several limitations in this study. As medical imaging technology have improved greatly during the study time span, the diagnosis sensitivity of primary spinal osseous tumors should lead to different prevalence in different time. Although the prognosis of spinal osseous tumors and longitudinal changes could not be fully assessed due to the nature of a retrospective study, we hope our work could provide useful epidemiological information as these data may have important implications for public health programs.

## Additional files

Additional file 1: **Table S1.** Treatment of primary spine osseous tumor. (DOCX 14 kb)
