# Supplementary material for: Epidemiological characteristics of primary spinal osseous tumors in Eastern China
Source: World J Surg Oncol. 2017 Apr 4;15:73. doi: 10.1186/s12957-017-1136-1 (PMC5379532; doi:10.1186/s12957-017-1136-1)
Supplement: Supplementary file 1 — Table S1. Treatment of primary spine osseous tumor. (DOCX 14 kb) [file 12957_2017_1136_MOESM1_ESM.docx]

**Table S1.** **Treatment of Primary Spine Osseous Tumors**

| Treatment | Malignant tumors | | Benign tumors | | Total | |
| --- | --- | --- | --- | --- | --- | --- |
|  | NO. | ％^1^ | NO. | ％^2^ | NO. | % |
| Surgery | 398 | 92.8％ | 187 | 24.0% | 585 | 48.4％ |
| Chemotherapy | 271 | 63.2％ | 0 | NA | 271 | 22.4％ |
| Radiotherapy | 34 | 7.9％ | 0 | NA | 34 | 2.8％ |
| Others | 6 | 1.4% | 35 | 4.5% | 41 | 3.4% |

%^1^: % in malignant tumors; %^2^: % in benign tumors; %: % in all tumors. NA：not applicable.
